# ROCK inhibitors beneficially alter the spatial configuration of TGFβ2-treated 3D organoids from a human trabecular meshwork (HTM)

**DOI:** 10.1038/s41598-020-77302-9

**Published:** 2020-11-20

**Authors:** Chiaki Ota, Yosuke Ida, Hiroshi Ohguro, Fumihito Hikage

**Affiliations:** Departments of Ophthalmology, Sapporo Medical University School of Medicine, Sapporo, Japan

**Keywords:** Ocular hypertension, Glaucoma, Eyelid diseases

## Abstract

To elucidate molecular pharmacology of Rho-associated coiled-coil containing protein kinase inhibitors (ROCK-i, Ripasudil and Y27632) on their efficiency for aqueous outflow, 2D or 3D cultures of a human trabecular meshwork (HTM) were prepared in the presence of TGFβ2. Those were examined by transendothelial electrical resistance (TEER, 2D), electronic microscopy (EM, 2D and 3D), expression of the extracellular matrix (ECM) including collagen1 (COL1), COL4 and COL6, and fibronectin (FN) by immunolabeling and/or quantitative PCR (3D), and solidity of 3D organoids by a micro-squeezer. TGFβ2 significantly increased the TEER values in 2D cultures, and the ECM expression indicated that the 3D organoids assumed a more densely packed shape. ROCK-i greatly reduced the TGFβ2-induced enhancement of TEER and the immunolabeled ECM expression of the 3D organoids. In contrast, the mRNA expression of COL1 was increased, and those of COL4 and FN were unchanged. EM revealed that TGFβ2 caused the HTM cells to become more compact and abundant ECM deposits within the 3D organoids were observed. These were significantly inhibited by ROCK-i. The dense solids caused by the presence of TGFβ2 were significantly suppressed by ROCK-i. Current study indicates that ROCK-i cause beneficial effects toward the spatial configuration of TGFβ2-induced HTM 3D organoids.

## Introduction

Glaucomatous optic neuropathy (GON), a progressive and chronic optic neuropathy that ultimately leads to irreversible blindness is known to be caused by several factors including the direct damage of axons, microvascular failure, genetic factors, structural failure from myopic change, autoimmunity, and others^1,2^. Among these causes, intraocular pressure (IOP) is recognized as the most important risk factor^3^. Thus, decreasing IOP by anti-glaucoma medication, laser treatment or surgery is the only evidence-based therapy that is currently available for the treatment of GON^4^. In the human eye, IOP levels are maintained by aqueous humor (AH) production and drainage through the trabecular meshwork (TM) in which approximately 70–90% of the AH is drained^5^. In terms of the mechanism responsible for the increase in IOP, elevated resistance to AH outflow from the TM caused by the excess deposition of extracellular matrix (ECM) such as collagens (COLs), fibronectin (FN) and others is primarily thought to be responsible for the development of both primary open angle glaucoma (POAG) and steroid-induced glaucoma (SG)^6^. In addition, the excess deposition of ECM has been reported to be mediated by elevated levels of TGFβ2 in AH in response to treatment with glucocorticoids^7^. In fact, it has been reported that TGFβ2 induces an increased resistance to in AH outflow, resulting an elevation in IOP in a rat model, and TGFβ2 treated TM cell cultures are extensively used in this research field^8^.

Rho-kinase (Rho-associated coiled-coil containing protein kinase; ROCK), a member of the serine-threonine protein kinase family, is involved in a variety of physiological functions, such as smooth muscle contraction, chemotaxis, neural growth, and others^9–11^. ROCK is composed of two isoforms, ROCK1 and ROCK2, which are distributed in various tissues including ciliary muscles, TM, the iris, and retina^12^. ROCKs are also involved in several types of pathogenesis including GON, several types of retinopathy, cataract, and corneal dysfunction^13–16^. Thus, ROCK is believed to be a promising therapeutic target for these diseases. In fact, ROCK inhibitors (ROCK-i) have been shown to reduce IOP in several animal models^17–19^, and Ripasudil hydrochloride hydrate (Rip), is now being used as a new type of anti-glaucoma drops for the treatment of POAG and ocular hypertension^20^. In addition, therapeutic possibilities by Rip will be also expected to corneal endothelial dystrophy and inflammation of retinal pigment epithelium^21,22^.

Some in vitro models using human TM (HTM) have recently been developed for use in analyzing the effects of TGFβ2 on changes in transcellular pressure and ease of outflow^8^. Using these models, the molecular mechanisms of pathological changes in glaucomatous TM as well as the efficacy of several anti-glaucoma medications including ROCK-i has been investigated^23^. Although most of these studies involved the use of conventional 2D cell cultures, despite the fact that the HTM is composed of multiple sheets in the human eye^24^, thus a relevant 3D cell culture model should be used in this research field. Our group recently established a 3D cell culture system using 3T3-L1 cells or human orbital fibroblasts (HOF) as disease models for thyroid associated orbitopathy (TAO)^25^, and the deepening of the upper eyelid sulcus (DUES) induced by prostaglandin analogues (PGs)^26,27^.

The purpose of the current study is to establish a physiologically relevant model by 3D cultures using HTM and TGFβ2, and to examine the effects of ROCK-i on the size, morphology and physical properties of the 3D organoids and their ECM expression.

## Results

**Figure 1:**
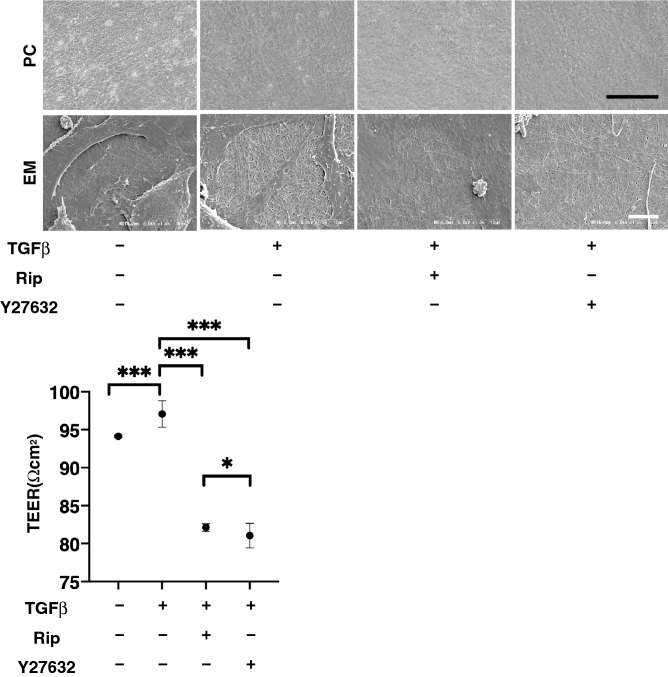
Representative phase contrast (PC) and electronic microscopic (EM) images (upper), transendothelial electrical resistance (TEER) (bottom) of 2D culture of HTM. Among the experimental groups treated with or without 5 ng/ml TGFβ2 in the presence or absence of 10 µM Ripasudil (Rip) or 10 µM Y27632, images by PC and EM of 2D cultures HTM at Day 5 are shown (upper panels). To evaluate barrier function (Ωcm^2^) of 2D cultured HTM monolayer, TEER (bottom) were measured. All data (n = 20, in each condition) are presented as arithmetic means ± standard error of the mean (SEM). N *P < 0.05, ***P < 0.005 (ANOVA followed by a Tukey’s multiple comparison test). Scale bar: PC; 50 µm, EM; 10 µm.

Morphology by phase-contrast (PC) and electronic microscopy (EM) show that the inter-cellar ECM network of 2D cultures of HTM cells at Day 6 was significantly increased upon exposure to a 5 ng/ml solution of TGFβ2 (Fig. 1, upper panels). Such TGFβ2-induced changes were markedly inhibited by the addition of either 10 µM Ripasudil (Rip) or Y27632. To evaluate the barrier function of these experimental groups of 2D cultured HTM cell monolayers, transendothelial electron resistance (TEER) was measured. Consistent with the morphological observations, the TEER values were significantly increase upon the administration of TGFβ2, and this change was diminished in the presence of either Rip or Y27632 (Fig. 1, lower panel).

**Figure 2:**
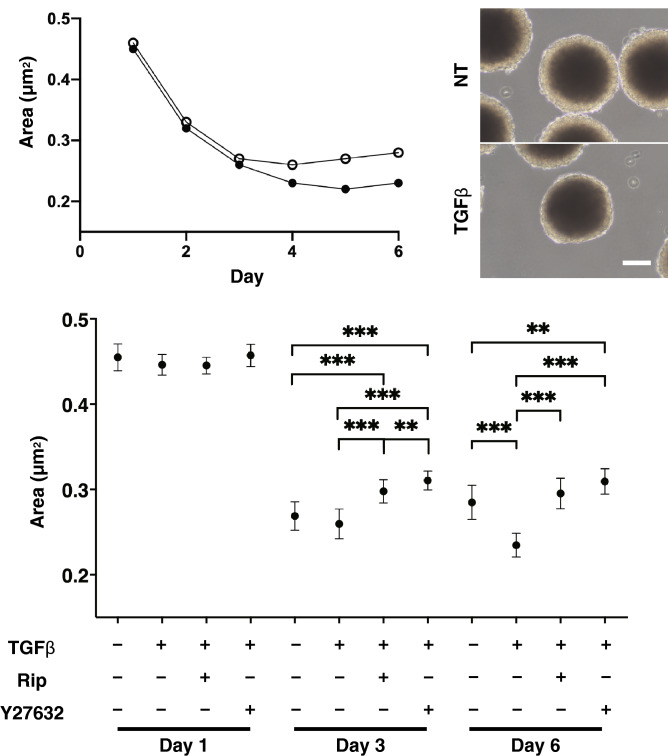
Change in HTM organoid size during their 3D culture in the presence or absence of TGFβ2, and their representative phase contrast (PC) images, and effects by Rock inhibitors toward the organoid sizes. Change of mean size of HTM organoids during 3D culture in the presence (TGFβ, closed circles) or absence (NT, open circles) of 5 ng/ml TGFβ2 are plotted up to Day 6 (upper left). Representative PC images of the 3D HTM organoids at Day 6 are shown (Scale bar: 100 µm, upper right). Among experimental groups treated with or without 5 ng/ml TGFβ2 in the presence or absence of 10 µM Ripasudil (Rip) or 10 µM Y27632, mean size of the 3D HTM organoids at Day 1, 3 and 6 are compared with each other. Data (n = 40 in each condition) are presented as arithmetic means ± standard error of the mean (SEM). **P < 0.01, ***P < 0.005 (ANOVA followed by a Tukey’s multiple comparison test).

Structurally, the TM in the human eye is known consist of multiple layers of sheets^24^. Thus, to investigate the physiological function and structure of TM, another suitable culture system would be desirable in terms of evaluating their three-dimensional (3D) properties. A 3D drop culture method^28^ was employed for this purpose. As shown in the upper panels of Fig. 2, uniform round-shape spheroidal 3D organoids were successfully generated from 20,000 HTM cells. In the process of maturing into the 3D organoid of control (NT), they gradually diminished in size, reaching a steady state by Day 6. Upon the administration of 5 ng/ml TGFβ2, the 3D organoid sizes became significantly smaller starting on Day 3 during the culture. In the presence of 10 µM Rip or Y27632, these TGFβ2-induced changes were significantly inhibited both at Day 3 and Day 6 (Fig. 2, lower panel).

**Figure 3:**
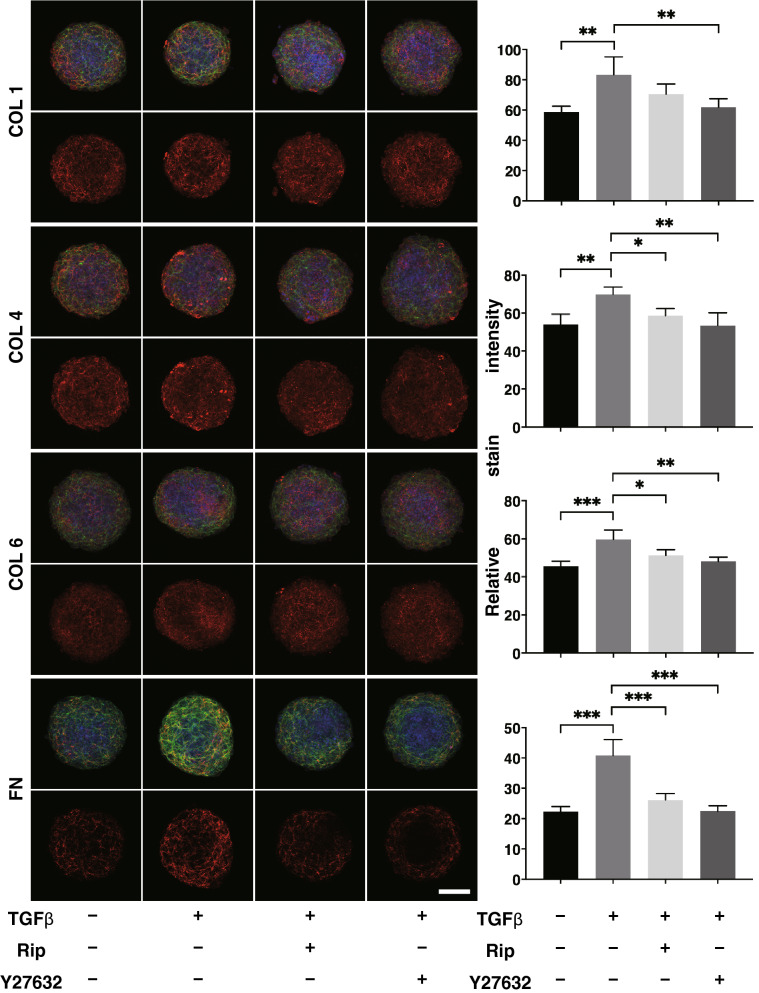
Immunofluorescence images of the expression of ECMs in HTM organoids. Among the experimental groups treated with or without 5 ng/ml TGFβ2 in the presence or absence of 10 µM Ripasudil (Rip) or 10 µM Y27632, 3D HTM organoids were immunostained by specific antibodies against ECMs (COL1, COL4, COL6 or FN, red), DAPI (blue) and phalloidin (green) in the left panels (Scale bar: 100 µm). The staining intensities are plotted in the right panels. All experiments were performed in duplicate using fresh preparations consisting of 10 organoids each. Data (total n = 20 different 3D organoids images in each experimental condition) are presented as the arithmetic mean ± standard error of the mean (SEM). *P < 0.05, **P < 0.01, ***P < 0.005 (ANOVA followed by a Tukey’s multiple comparison test).

**Figure 4:**
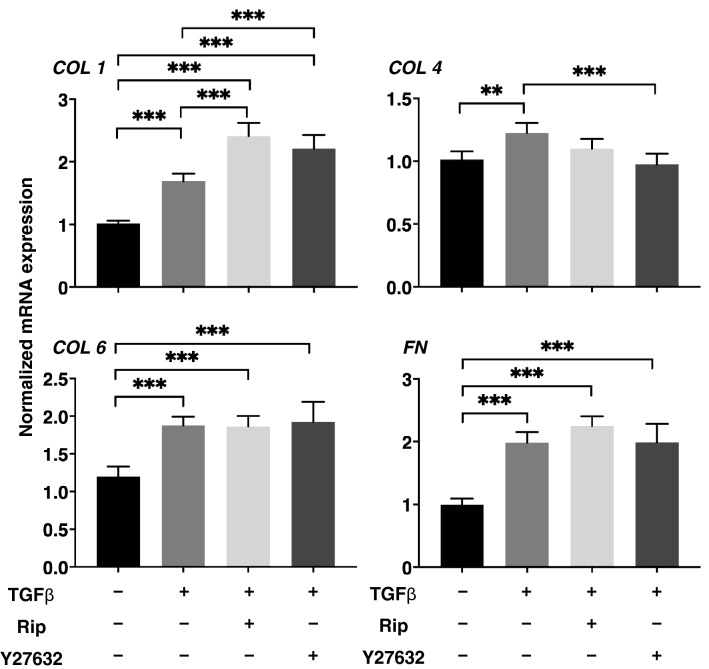
mRNA expression of ECMs in HTM organoids. Among the experimental groups treated with or without 5 ng/ml TGFβ2 in the presence or absence of 10 µM Ripasudil (Rip) or 10 µM Y27632, 3D HTM organoids at Day 6 were subjected to qPCR analysis to estimate the expression of mRNA in ECMs (*COL1*, *COL4*, *COL6* or *FN*). All experiments were performed in duplicate using fresh preparations consisting of 16 organoids each. Data (n = 5 in each condition) are presented as arithmetic means ± standard error of the mean (SEM). **P < 0.01, ***P < 0.005 (ANOVA followed by a Tukey’s multiple comparison test).

**Figure 5:**
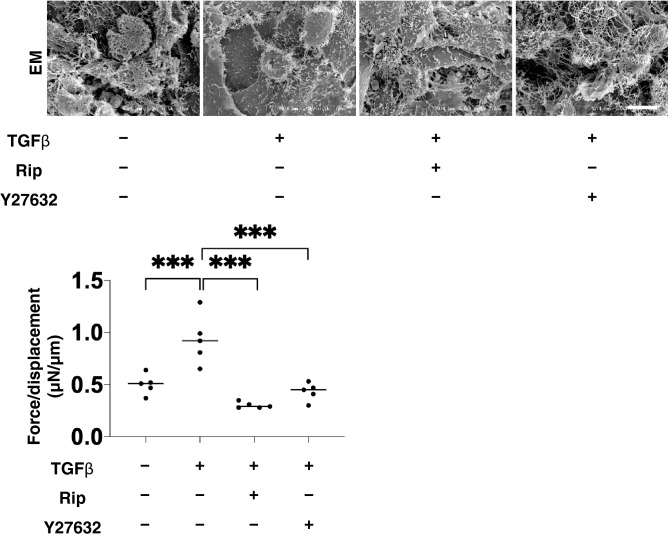
Ultrastructure and physical solidity of 3D HTM organoids. Among the experimental groups treated with or without 5 ng/ml TGFβ2 in the presence or absence of 10 µM Ripasudil (Rip) or 10 µM Y27632, ultrastructure and physical solidity of their 3D organoids at Day 6 were analyzed by electron microscopy (upper) and a micro-squeezer (μN/μm force/displacement, bottom), respectively. Data (n = 5 in each condition) are presented as arithmetic means. Scale bar: 10 µm. ***P < 0.005 (ANOVA followed by a Tukey’s multiple comparison test).

To elucidate the mechanism involved in the differences in these 3D organoid sizes, the expression of the major ECM framing the 3D organoids including collagen1 (COL1), COL4, COL6, and fibronectin (FN) were evaluated by immunocytochemistry (Fig. 3) and quantitative PCR (Fig. 4). Upon administering a 5 ng/ml solution of TGFβ2, the levels of both the immunofluorescence labeling and mRNA expression of all four ECMs were significantly enhanced (Figs. 3 and 4). In the presence of 10 µM Rip or Y27632, this enhanced immunofluorescence labeling of all four ECMs by TGFβ2 was marked suppressed (Fig. 3). Similarly, quantitative qPCR analysis indicated that the TGFβ2-induced changes in COL4 were also inhibited by the presence of 10 µM Rip or Y27632 (Fig. 4). While, in contrast, those in COL1 were further enhanced, and the changes in COL6 and FN were not significantly affected (Fig. 4). We conclude that the discrepancy between mRNA expression and the immunohistochemistry labeling of these ECMs were as follows; 1) TGFβ2 significantly increased the expression of ECMs resulting in the shape of the 3D organoid becoming more densely packed, 2) Rip or Y27632 had no significant influence on the TGFβ2-induced enhancement of the mRNA expression of ECMs except COL1, 3) These changes in ECM expression could have altered the spatial 3D configuration of the 3D organoid, this influencing the levels of immunohistochemistry labeling. This rationale is supported by the ultrastructure of the 3D HTM organoids, as shown by EM, and their physical property based on a micro-squeezer analysis (Fig. 5). Upon the administration of TGFβ2, each HTM cell was located closer and their ECM deposits were much more abundant as compared to the control. In the presence of 10 µM Rip or Y27632, wider inter-cellular intervals and less ECM deposits within 3D organoid surface were observed (Fig. 5, upper panel). Concerning the physical solidity of the 3D organoid, the force (µN) required to cause deformity of the 3D organoid until the diameter became half by mechanical compression by micro-squeezer analysis (Supplementary Video 1). As shown in the lower panel of Fig. 5, much higher forces were required when TGFβ2 was administered as compared to control. In the presence of 10 µM Rip or Y27632, these TGFβ2-induced effects were significantly suppressed to levels nearly comparable to control.

## Discussion

It is well known that there are two major AH outflow pathways, namely, the conventional pathway through TM and the uveoscleral pathway that contribute to the homeostasis of AH dynamics and IOP maintenance^29^. Understanding these complex mechanisms for these dual AH outflows is extremely important for the etiology of GON and its therapeutic strategy including anti-glaucoma medications. Therefore, to study the pharmacokinetics of anti-glaucoma medications, methods to separate the conventional and uveoscleral pathway are needed. A numbers of studies using conventional 2D cell cultures of TM have been performed to achieve this^30,31^. However, since it is well known that HTM is comprised of multiple sheet structures^24^, the use of some advanced cell culture techniques allowed us to enable the 3D construction of HTM.

The 3D organoid culture system has recently received more attention concerning its possible use as a disease model through an ex vivo approach^28^. As compared to the conventional 2D culture system, this 3D culture method can be used to evaluate the structure of tissues including the biological features surrounded by the network of ECM proteins^32^. However, in contrast, the 3D system has some disadvantages in terms of its inabilities to determine the quantity of the target protein because of the 3D organoid is composed smaller amounts of cells in addition to the several technical difficulties associated with this 3D culture^28,33^. In a previous study, Torrejon et al.^34^ developed a 3D in vitro model using scaffolds that were fabricated using standard photolithographic techniques and found that the overexpression of ECM, impaired HTM cell phagocytic activity and increased the outflow resistance in the case of SG. Thus, they concluded that in 3D cultures a more accurate regulation of the apoptosis trigger and cell adaptation mechanisms was detected than in 2D models. In fact, Vernazza et al.^35^ compared between the 2D and 3D in vitro models of HTM in regard to cellular responses after chronic stress exposure, and demonstrated that 3D cultured cells are more sensitive to intracellular reactive oxidative specie production induced by hydrogen peroxide treatment as compared to a conventional 2D culture. In line with these findings, the 3D HTM model shows the ability to better mimic the in vivo cell behavior in adaptive responses to chronic oxidative stress than 2D. However, since HTM is known to be composed of unique multiple layer sheets, 3D cultured HTM cells may not perfectly replicate such real construction of the HTM, but 3D culture of the HTM still have great advantages as above in comparison to the conventional 2D culture method. Furthermore, these 3D culture techniques may have some limitations in terms of mimicking the physiological and pathological conditions because of the presence of an unnecessary scaffolds. Alternatively, our group recently developed a 3D cell drop culture system in which no scaffold is required^25–27^. Using this technique, we were able to obtain 3D organoids from HOF in patients with TAO using a drop culture method, and found that HIF2A played a critical role in mediating LOX-dependent ECM accumulation^25^. More recently, to elucidate the molecular etiology of DUES induced by PGs, we employed this 3D culture system using HOF and 3T3-L1 cells, and found that PGs significantly suppressed 3D organoid sizes, and modulated the spatial distribution of several ECMs surrounding the 3D organoids^26,27^. We therefore concluded that our developed 3D cell culture would be suitable for investigating the molecular etiology of TAO and DUES and also will be applicable for use in many other cells and organs.

It has been reported that ROCK contributes to the actin cytoskeleton and fibronectin matrix assembly^23,36,37^, and ROCK-i alter cytoskeletal arrangement and cell shape in TGFβ2 treated HMT cells^38,39^. It therefore appears that these ROCK-i postulated to reduce TM stiffening eliciting an increase in the ease of AH outflow, resulting in a significant decrease in IOP levels. To study the effects of ROCK-i on HTM, we utilized corresponding 3D cultures of HTM and found the following observations: (1) TGFβ2 significantly increased TEER in 2D cultures, and the expression of ECMs resulted in more packed 3D organoids, (2) ROCK-i greatly reduced the TGFβ2-induced enhancement of TEER and ECM expression of 3D organoids, (3) upon the administration of ROCK-i, the mRNA expression of COL1 was specifically increased, (4) EM showed that TGFβ2 caused HTM cells to become in closer proximity and abundant ECM deposits within the 3D organoid were observed. These changes were significantly inhibited by ROCK-i, and (5) TGFβ2 induced a significant increase in the hardness of the 3D organoids, the effects of which were diminished by the presence of ROCK-i.

ECM is an important multifunctional molecular group, which is involved in structurally supporting organs as well as to modulate cell–cell signals and regulate various cellular functions^40^. COLs consist of more than 30 subspecies of triple helical family proteins^41^. Among them the most abundant are COL1. COL4 is a major component of the basement membrane (BM)^41–43^. COL6 forms microfibrils between BM and thick bundles of COL1^44^. Furthermore, COL6 is also thought to be an important regulator in several cellular functions such as adipogenesis^45^. FN exists in a functional form that is composed of highly interwovened fibers, and is the key molecule defining cell shape and contractility in association with COL1^46^. In TM cells, TGFβ2 is known to activate cytoplasmic Smad2/3^47,48^, which, in turn, leads to an increased expression of ECMs, such as FN and COL4 as fibrotic changes. These changes can explain the TGFβ2-induced impediment of AH outflow through the TM resulting in elevated IOP levels^49^. In regards to ROCK-i toward such TGFβ2-induced changes within HTM, a significant suppression of COL4 and FN were reported in a study using a scaffold assisted 3D HTM model^34^. However, in the present study, although the mRNA expression of COL1 and COL4 was increased or suppressed by the presence of ROCK-i, the protein expression of all ECMs (COL1, COL4, COL6, and FN), as evidenced by immunocytochemistry data, were inhibited. Similar observation of the discrepancy between mRNA expression and immunostaining intensities of the ECM was also recognized in our previous study related to DUES model using 3T3-L1 3D organoid^26^. As a possible reason, we speculated that spatial localization within the 3D organoids in addition to the abundance of each molecule may have been involved. In fact, in our previous report, we found that remarkable difference in trypsin sensitivity between 2 and 3D cell culture from 3T3-L1 cells, that is, the 2D cells were rapidly dispersed within few minutes in the presence of 0.2% trypsin, whereas 3D organoid was almost resisted against 0.2% trypsin until 12hrs^26^. Since COL1 is the major ECM protein that provides the framework necessary to sustain the structure in a variety of cells with COL4 within BM^44^, it is possible that such changes in the gene expressions of *COL1* and *COL4* would induce changes in spatial 3D configuration caused by the ROCK-i. These speculations were easily visualized by ultrastructure observations by EM and micro-indentation by means of a micro-squeezer as shown in Fig. 5. The latter analysis is only possible when our 3D drop culture method is used because this permits a single living 3D organoid to be observed. Thus, our 3D cell culture model appears to more closely recapitulate the ultrastructure and physiological functions of HTM.

In conclusion, our newly developed 3D cell culture method permitted a better understanding of the molecular pharmacology of ROCK-i toward TGFβ2 treated HTM, a common model of POAG. Previously, Kaneko et al.^50^ described interesting observations of effects of Rip and Y27632 toward HTM cells and Schlemm’s canal endothelial cells. Furthermore, it has been identified that dexamethasone also induced similar effects as TGFβ2 toward HTM cells. However, most of these studies accomplished using a conventional 2D cell culture method. Therefore, the investigation of these study subjects using our new 3D culture method will be our next projects.

## Materials and methods

### Chemicals and drugs

Dulbecco’s Modified Eagle’s Medium (DMEM) (# 11965092, Gibco/Thermo Fisher Scientific, Waltham, MA), fetal bovine serum (FBS) (# 16-000-044, Gibco/Thermo Fisher Scientific), L-glutamine (# 25030081, Gibco/Thermo Fisher Scientific), antibiotic/antimycotic (# 15240062, Gibco/Thermo Fisher Scientific), penicillin/streptomycin (# 15140122, Gibco/Thermo Fisher Scientific), Ficoll-Paque Plus (# 17-1440-03, GE Healthcare, Piscataway, NJ), Puromycin (# P8833, Sigma-Aldrich, St Louis, MO), Methocel A4M (# 94378, Sigma-Aldrich), TGFβ2 (2.5 ng/mL in 4 mM HCL, R&D systems, Minneapolis, MN), Ripasudil (Rip) (provided by Kowa Company Ltd., Nagoya, Japan), Y27632 (Sigma-Aldrich, St Louis, MO).

### Preparation of 3D organoid cultures of human trabecular meshwork (HTM) Cells

Commercially available the human trabecular meshwork (HTM) immortalized by transfection with an original defective mutant of the SV40 virus (Applied Biological Materials Inc., Richmond Canada) was used in this study. The HTM cells were grown in 150 mm 2D culture dishes until reaching 90% confluence at 37 °C in grown medium A composed of HG-DMEM containing 10% FBS, 1% L-glutamine, 1% antibiotic–antimycotic and were maintained by changing the medium every other day. All studies were conducted using cells up to 20th passage. HTM cells prepared as above were further processed for 3D organoid preparation or transendothelial electron resistance (TEER) experiment described below.

The 3D organoids of HTM were generated by a hanging droplet spheroid three-dimension (3D) culture system as described in a previous report^25^. Briefly, 90% confluence HTM cells in 150 mm 2D culture dishes as above were washed with phosphate buffered saline (PBS), and the cells were detached using 0.25% Trypsin/EDTA. After centrifugation for 5 min at 300×*g*, the cell pellet was re-suspended in organoid medium A composed of grown medium A supplemented with 0.25% methylcellulose (Methocel A4M) to facilitate stable 3D organoid morphology. 20,000 HTM cells in the 28 μL of organoid medium A were placed into each well of the hanging drop culture plate (# HDP1385, Sigma-Aldrich) (Day 0). At Day 1, 5 ng/mL TGFβ2 was added in the absence or presence of 10 µM Rip or 10 μM Y27632 to organoid medium A in each experimental group (organoid medium B). The dosage of these ROCK-i was according to the previous study using a different way of the 3D cell culture of HTM and Y27632^34^. On every following day, 14 μL of organoid medium B was removed and a fresh14 μL portion of organoid medium B was added to each well. As controls, the HTM cells were maintained as above with vehicle organoid medium A. 3D organoid cultures as above were maintained until Day 6.

### Transendothelial electrical resistance (TEER) measurement and scanning electron microscopy analysis of 2D HTM culture

HTM cell monolayer TEER was determined according to previously described methods^50^. Briefly, HTM cells prepared in 150 mm 2D cultured dishes as above were washed with a PBS, and the cells were detached using 0.25% Trypsin/EDTA. After centrifugation for 5 min at 300×*g*, the cell pellet was re-suspended in grown medium A and HTM cells were seeded on 12 well plates for TEER (0.4 μm pore size and 12 mm diameter; Corning Transwell, Sigma-Aldrich) at a density of 2.0 × 10^4^ cells per well. In each well of the TEER plate, the apical side (inside of the membrane inserts) and basal side (outside of the membrane inserts) were maintained in 0.5 mL and 1.5 mL of growth medium A, respectively. When cells had reached approximately 80% confluence, 5 ng/mL TGFβ2 was added to the grown medium A of the apical side in the absence or presence of 10 µM Rip or 10 μM Y27632 (Day 1). This culture medium of the apical side in each experimental group was changed every other day. At Day 6, TEER (Ωcm^2^) was measured using an electrical resistance system (KANTO CHEMICAL CO. INC., Tokyo, Japan) according to the manufacturer’s instructions after washing twice with PBS. Alternatively, after washing with PBS as above, HTM cells on the membrane were processed by scanning electron microscopy (EM) using HITACHI S-4300 microscope operated at 5 keV (the detector features 1280 × 960 pixel).

### Real time PCR analysis of ECM genes

Isolated RNA from 2D or 3D cultured HTM using a RNeasy mini kit (Qiagen, Valencia, CA) were processed for reverse transcription by the SuperScript IV kit (Invitrogen) as according to the manufacturer’s protocols. Then, real-time PCR was performed using the Universal Taqman Master mix by a StepOnePlus system (Applied Biosystems/Thermo Fisher Scientific). The cDNA levels shown as fold-change relative to the control after normalization using the 36B4 (*Rplp0*).

### Sequences of primers and Taqman probes

**human RPLP0**

- (Probe: 5′-/56-FAM/CCCTGTCTT/ZEN/CCCTGGGCATCAC/3IABkFQ/-3′),
- (Forward: 5′-TCGTCTTTAAACCCTGCGTG-3′),
- (Reverse: 5′-TGTCTGCTCCCACAATGAAAC-3′).

**human COL1A1**

- (Probe: 5′-/56-FAM/TCCAGGGCC/ZEN/AAGACGAAGACATC/3IABkFQ/-3′),
- (Forward: 5′-GACATGTTCAGCTTTGTGGAC-3′),
- (Reverse: 5′-TTCTGTACGCAGGTGATTGG-3′).

**human COL4A1**

- (Probe: 5′-/56-FAM/TCATACAGA/ZEN/CTTGGCAGCGGCT/3IABkFQ/-3′),
- (Forward: 5′-AGAGAGGAGCGAGATGTTCA-3′),
- (Reverse: 5′-TGAGTCAGGCTTCATTATGTTCT-3′).

**human COL6A1**

- (Forward: 5′-CCTCGTGGACAAAGTCAAGT-3′),
- (Reverse: 5′-GTGAGGCCTTGGATGATCTC-3′).

**human FN1**

- (Forward: 5′-CGTCCTAAAGACTCCATGATCTG-3′),
- (Reverse: 5′-ACCAATCTTGTAGGACTGACC-3′).

### Image acquisition and analysis of 3D HTM organoids

Bright field images of each organoid were obtained in 4 × objective lenses using inverted microscope (Nikon ECLIPSE; Tokyo, Japan). The largest cross-sectional area was calculated using NIH ImageJ software version 1.52p (https://imagej.net/Fiji/Downloads).

### Immunocytochemistry of 3D HTM organoids

Immunocytochemistry of 3D HTM organoids was conducted by the method described recently^26,27^. All procedures were performed at RT unless otherwise stated. Briefly, 3D HTM organoids obtained at Day 6 as above under several conditions were fixed in 4% PFA in PBS overnight, blocked in 3% BSA in PBS for 3 h, washed twice with PBS for 30 min. Thereafter those were incubated with an anti-human COL1, COL4, COL6 or FN rabbit antibody (1:200 dilutions) at 4 °C overnight. After washing 3 times with PBS for 1 h each, the specimens were then incubated with 1:1000 dilutions of a goat anti-rabbit IgG (488 nm), phalloidin (594 nm) and DAPI for 3 h followed by being mounted with ProLong Gold Antifade Mountant with a cover glass. The serials-axis 2.2 μm interval immunofluorescent images during a z-plane between 35 μm from their surface were obtained by a Nikon A1 confocal microscopy using a × 20 air objective with a resolution of 1024 × 1024 pixels and were converted as Z-stack image using the maximum intensity projection feature of NIS element 4.0 software (Nikon; Tokyo, Japan, https://www.microscope.healthcare.nikon.com/products/software/nis-elements). For analysis of their signal intensity was calculated using NIH Image J software version 1.52p. Signal intensity of organoids was expressed as intensity/surface area measured at 35 μm from the top of the organoid in the z-plane. The surface area was calculated as follows: surface area = D × A/(A + π × H2), where D (μm) indicates organoid diameter, A (μm^2^) indicates area of sectioned organoid, and H (μm) indicates height (= 35 μm).

### Solidity measurement of 3D organoid

The solidity of organoids was measured using micro-squeezer (MicroSquisher, CellScale, Waterloo, ON, Canada) equipped with a microscale compression system composed by a 406 μm diameter cantilever as recently reported^25^. A single organoid placed on a 3-mm × 3-mm plate was compressed to 50% deformation, as determined by a microscopic camera, for 20 s. The force requiring 50% strain was measured through the cantilever, and data are expressed as force/displacement (μN/μm).

### Statistical analysis

All statistical analyses were performed using Graph Pad Prism 8 (GraphPad Software, San Diego, CA). For comparison of two mean values, a two-tailed Student’s t-test was used to calculate statistical significance with a confidence level greater than 95%. To analyze the difference in groups, a grouped analysis with two-way analysis of variance (ANOVA) followed by a Tukey’s multiple comparison test was performed. Data are presented as arithmetic means ± standard error of the mean (SEM).

## Supplementary information

Supplementary Legend.

Supplementary Video 1.
