# Supplementary material for: ROCK inhibitors beneficially alter the spatial configuration of TGFβ2-treated 3D organoids from a human trabecular meshwork (HTM)
Source: Sci Rep. 2020 Nov 20;10:20292. doi: 10.1038/s41598-020-77302-9 (PMC7680137; doi:10.1038/s41598-020-77302-9)
Supplement: Supplementary file 1 — Supplementary Legend. [file 41598_2020_77302_MOESM1_ESM.docx]

**Supplemental movie 1**

A single 3D HTM organoid placed on a plate was compressed to 50 % deformation during 20 sec. These processes were continuously monitored by a microscopic camera.
